# Determinants of image quality in transthoracic echocardiography: A retrospective cohort study

**DOI:** 10.1177/20480040261445490

**Published:** 2026-04-21

**Authors:** Aradhai Bana, Ciaran Grafton-Clarke, Rui Li, Zia Mehmood, Sunil Nair, Bahman Kasmai, Simon Eccleshall, Alisdair Ryding, Timothy Gilbert, Samer Alabed, Andrew J Swift, Peter Swoboda, Vassilios S Vassiliou, Gareth Matthews, Pankaj Garg

**Affiliations:** 1 Norwich Medical School, 6106 University of East Anglia, Norwich, UK; 2 6106 Cardiology Department, Norfolk and Norwich University Hospitals Foundation Trust, Norwich, UK; 3 Division of Clinical Medicine, Immunity & Cardiovascular Disease, 7315 University of Sheffield, Sheffield, UK; 4 National Institute for Health and Care Research, Sheffield Biomedical Research Centre, Sheffield, UK; 5 Leeds Institute of Cardiovascular and Metabolic Medicine, 4468 University of Leeds, Leeds, UK

**Keywords:** Echocardiography, image quality, cost-benefit analysis, health care costs

## Abstract

**Objectives:**

To identify patient factors associated with poor transthoracic echocardiographic image quality and to evaluate whether a simple pre-test triage model could improve imaging efficiency.

**Design:**

Retrospective cohort study with derivation and independent validation cohorts, together with a model-based cost-effectiveness analysis.

**Setting:**

Single large UK tertiary centre using routinely collected data from 2010 to 2020.

**Participants:**

70,597 adult transthoracic echocardiograms, divided into a derivation cohort (n = 40,000) and an independent validation cohort (n = 30,597).

**Main outcome measures:**

Poor image quality (limited or non-diagnostic vs good or adequate), model discrimination, sensitivity, specificity and comparative imaging costs.

**Results:**

Of 70,597 studies, 24,213 (34.3%) were poor quality, including 8582 (12.2%) non-diagnostic and 15,631 (22.1%) limited studies. Lung disease was the strongest predictor (OR 2.04, 95% CI 1.59 to 2.61), followed by suspected heart failure, inpatient status, arrhythmia, prior cardiac surgery and permanent pacemaker (all *p* < 0.01). Validation performance was modest (AUC 0.58; sensitivity 67.3%; specificity 45.7%). In a model-guided simulation using 2024/25 NHS tariffs, total imaging costs were lower than with standard care (£11.85 million vs £12.16 million), yielding an estimated saving of £317,331.

**Conclusions:**

Several routinely available clinical factors influence transthoracic echocardiographic image quality. Although individual-level prediction is modest, pre-test triage may help direct higher-risk patients to contrast echocardiography or alternative imaging, reducing repeat testing and improving efficiency.

## Introduction

**Figure 1. fig1-20480040261445490:**
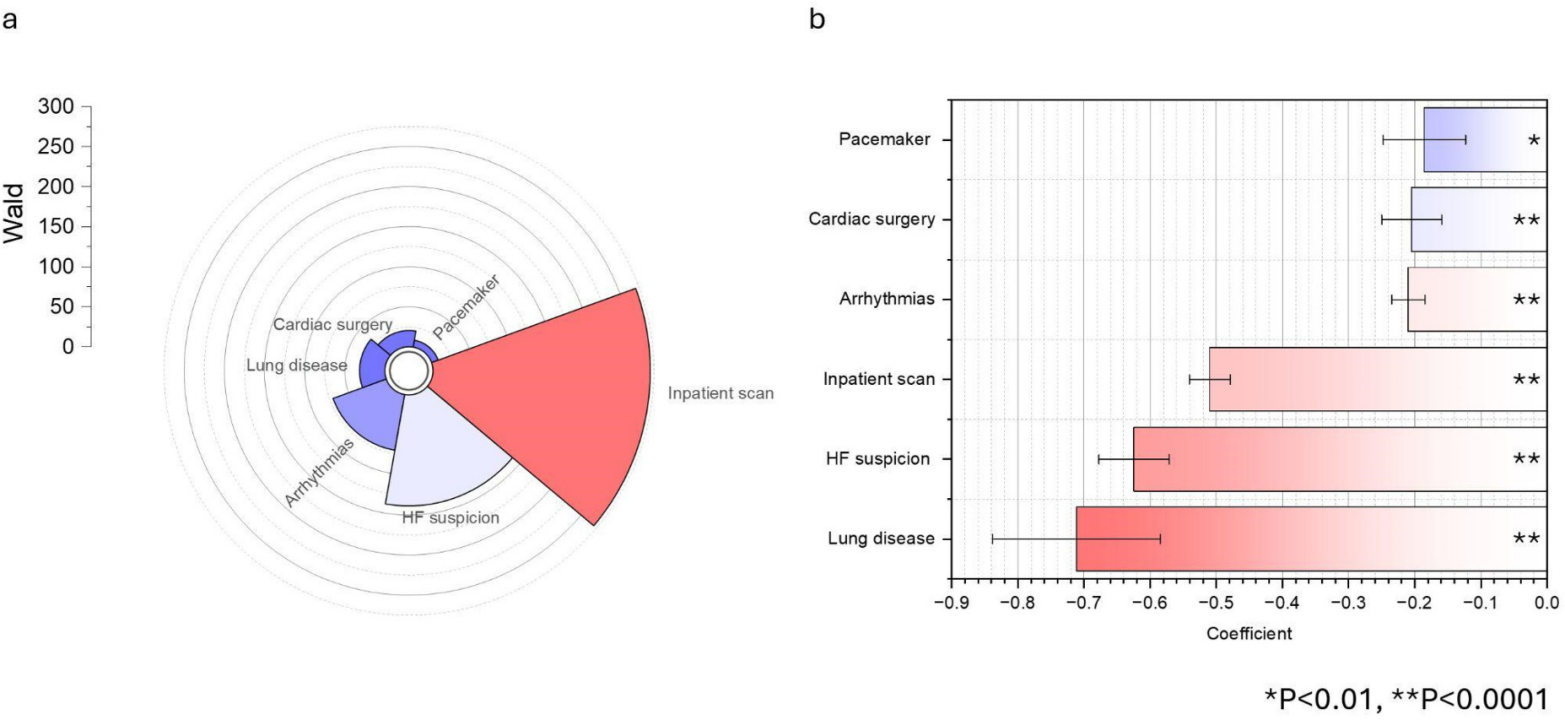
Logistic regression-derived coefficients and odds ratios (ORs) for predictors associated with nondiagnostic echocardiogram image acquisition. Panel (a) displays Wald statistic values (radial bars indicate the magnitude of the test statistic for each risk factor, with warmer shading denoting a larger effect size). Panel (b) presents corresponding ORs with 95 % confidence intervals and p-values (*p < 0.01; **p < 0.0001). Red shading denotes factors most strongly associated with decreased diagnostic quality. Error bars indicate the 95 % CI. HF = heart failure. HF: heart failure.

**Figure 2. fig2-20480040261445490:**
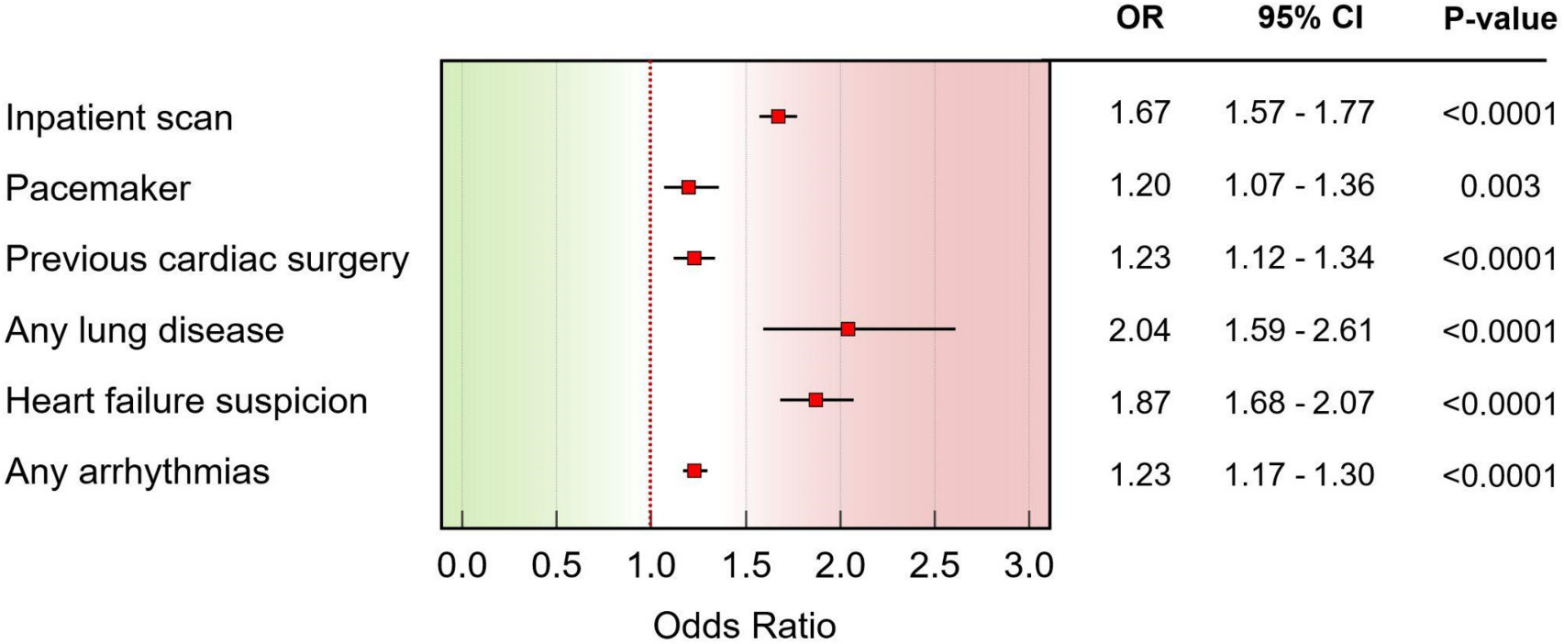
Forest plot illustrating the odds ratios (ORs) of patient-related clinical factors predictive of nondiagnostic echocardiographic images. The red boxes denote the point estimates for each risk factor, while horizontal lines represent the corresponding 95% confidence intervals (CI). The vertical dashed line at OR = 1.0 indicates neutrality. Factors positioned to the right of this line are significantly associated with increased odds of nondiagnostic images. Statistical significance (p-values) is indicated to the right. OR: odds ratio; CI: confidence interval.

**Figure 3. fig3-20480040261445490:**
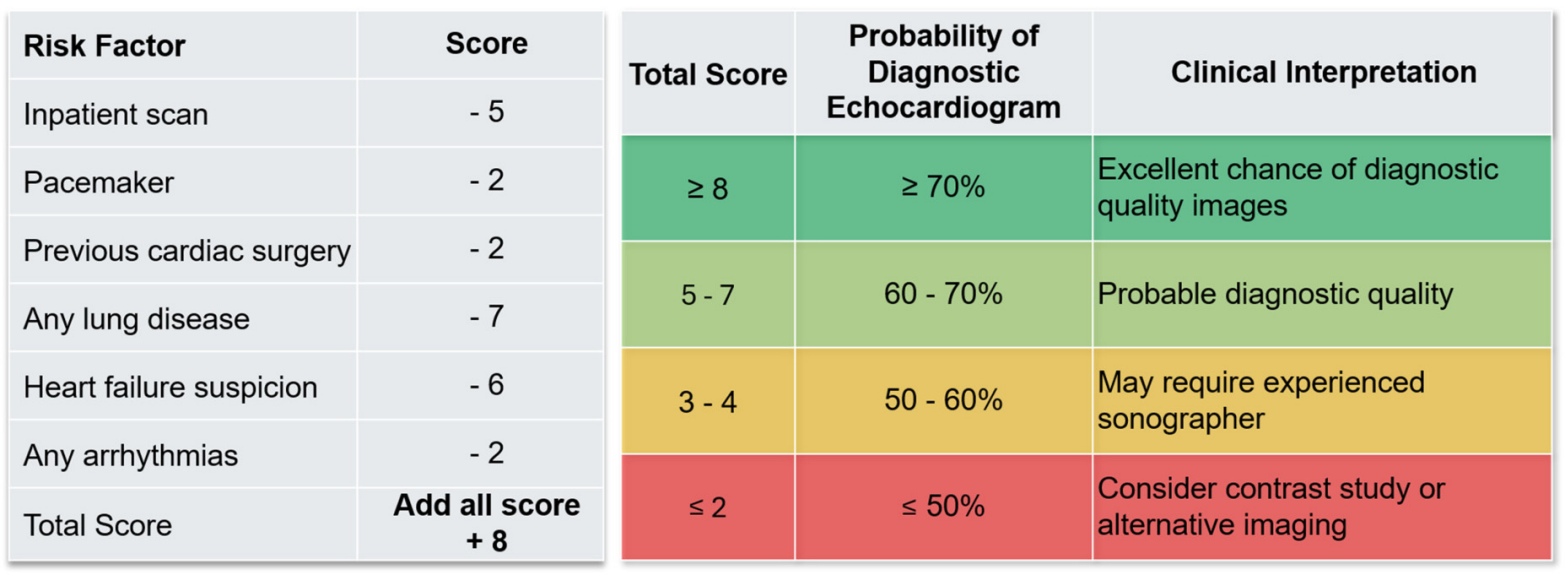
Clinical scoring tool for predicting the diagnostic quality of echocardiographic images based on patient-related risk factors. Panel (a) assigns negative scores to specific clinical factors influencing echocardiogram quality, generating a cumulative score (with baseline adjustment of +8). Panel (b) stratifies total scores into probability categories for diagnostic image acquisition and provides corresponding clinical interpretations, guiding decision-making for imaging modality selection.

Cardiovascular disease (CVD) remains a leading cause of morbidity and mortality globally, with more than 18.6 million deaths reported in 2019.^1^ This is a sharp increase since 1990, and while the age-standardised mortality has declined over the years, the absolute number of cases has only risen.^2^ In the US, CVD accounted for 1 in 5 deaths in 2021, and the American Heart Association reports that the burden of disease by CVD continues to increase even now.^3^[Fig fig1-20480040261445490][Fig fig2-20480040261445490][Fig fig3-20480040261445490]

Echocardiography has become the primary cardiovascular imaging modality due to its versatility, safety, cost-effectiveness, and widespread availability. It remains the first-line diagnostic test for most patients presenting with cardiac symptoms or suspected cardiac disease, including heart failure, valvular disorders, structural heart disease, and pericardial conditions.^4–6^ Globally, the number of echocardiographic examinations performed annually has risen significantly. In the United States alone, approximately 7.1 million echocardiograms are conducted each year,^7,8^ with an increase in use predicted in the UK due to the ageing population and changing cardiovascular epidemiology.^9^

Despite the extensive clinical utility of echocardiography, a significant proportion of studies yield non-diagnostic or limited-quality images, resulting in repeated imaging, increased healthcare costs, and inefficiencies within healthcare systems.^10,11^ Previous observational studies report that nearly 17.7% of hospital admissions undergo echocardiography, and among these patients, approximately 38% have repeated imaging within one year, often with minimal incremental diagnostic value.^12^ These redundant imaging studies do not significantly influence clinical management or patient outcomes.^13^ Early evidence suggests that targeted imaging strategies, such as point-of-care ultrasound or handheld echocardiography screening, may safely reduce unnecessary comprehensive echocardiograms by almost 60% in cases where echocardiograms are rarely indicated without adversely affecting patient outcomes.^14^ Therefore, an in-depth understanding of the variables influencing the quality of echocardiographic imaging could inform the implementation of a triage system based on risk-factor profiles, which could help address these inefficiencies.

We hypothesised that a clinical prediction model based on routinely collected patient characteristics could identify echocardiographic studies likely to be of inadequate diagnostic quality. The objective of this study was to develop and validate such a predictive model using data from a large single-centre echocardiography database. The overarching goal is to enhance imaging efficiency, reduce unnecessary repeat imaging, and optimise resource utilisation.

## Materials & methods

### Study design and population

This study was an analysis of historically collected data from a large, single-centre, tertiary cardiac centre (Norfolk and Norwich University Hospital, Norwich, UK). The study cohort was derived from a comprehensive clinical echocardiography database, encompassing all consecutive adult patients who underwent transthoracic echocardiography for standard clinical indications between January 2010 and December 2020. This retrospective cohort study is reported with reference to the STROBE statement for observational studies.

### Regulatory framework and ethical considerations

The investigation was conducted in conformity with the principles outlined in the Declaration of Helsinki. The project was established as a formal quality improvement initiative in the Cardiology Department (Card_2020-21_a08). It was approved by the local Ethics Committee (Ref: 2020/21-075, UEA). Following the United Kingdom's Research Ethics Committee framework for service evaluation, specific informed consent from individual patients was not required for this analysis. The study exclusively involved the analysis of a pre-existing, pseudonymised dataset, where all clinical and imaging parameters had been acquired as an integral component of standard diagnostic care.

All data were maintained and analysed within the secure NHS digital environment, ensuring adherence to the General Data Protection Regulation and the Data Protection Act 2018.

### Echocardiographic data acquisition

Most transthoracic echocardiograms were performed by British Society of Echocardiography (BSE)–accredited sonographers, who supervise the departmental practice. All studies followed a standardised institutional protocol closely aligned with BSE recommendations. Patient height, weight, and cardiac rhythm were recorded. Comprehensive datasets, including standard parasternal, apical, and subcostal views, were acquired for each patient. All imaging parameters and measurements were recorded at the time of the scan into a secure clinical imaging database. Operator identifiers, patient-positioning details, ultrasound machine vendor or software, transducer selection and advanced imaging settings were not consistently available in the historical database and therefore could not be modelled.

### Definition of image quality

At the time of clinical reporting, sonographers recorded overall image quality using routine departmental categories: good, adequate, limited or non-diagnostic. For the primary analysis, good and adequate studies were grouped as diagnostically acceptable, whereas limited and non-diagnostic studies were grouped as poor quality. A limited study retained partial diagnostic utility but did not permit complete assessment using standard views; a non-diagnostic study did not permit reliable interpretation of the clinical question.

### Data curation

Data were curated through a rigorous cleaning process. The clinical setting for each scan was recorded and categorised as either inpatient or outpatient. Studies were excluded if key data fields, specifically patient gender and the sonographer-reported image quality, were not recorded. For all included patients, age was calculated based on the date of the indexed scan. The recorded cardiac rhythm for each patient was dichotomised for analysis into sinus rhythm or non-sinus rhythm (atrial fibrillation, atrial flutter, or paced rhythm). This process resulted in a final analytical cohort of 70,597 patients for whom complete demographic, clinical, and imaging quality data were available for analysis.

Furthermore, key clinical variables were extracted from the free text “indication for scan” section of the echocardiography report. This allowed for the classification of patients based on the presence of major comorbidities and clinical indications, including: active smoking, diabetes mellitus, hypertension, history of myocardial infarction, heart failure, prior cardiac surgery, prior lung disease, transcatheter aortic valve implantation, pericardial effusion, reported breathlessness, congenital heart disease (including repaired Tetralogy of Fallot and atrial septal defect), cardiac transplantation, aneurysm repair, significant valvular heart disease (aortic regurgitation, aortic stenosis, mitral regurgitation, mitral stenosis, or prior mitral valve repair), and obstructive sleep apnoea. For the present analysis, lung disease was treated as a pragmatic binary variable derived from the free-text indication when any pulmonary comorbidity was explicitly documented; subtype and severity were not consistently available and therefore could not be modelled separately.

To achieve this systematically, a bespoke natural language processing (NLP) pipeline was developed in Python. This rule-based text-mining algorithm first performed semantic segmentation to isolate the “indication for scan” text from the remainder of the report. Subsequently, this targeted text was interrogated using a regular expression engine against a predefined lexicon of clinical terms, abbreviations, and their variants. The detection of a specific term automatically generated a corresponding binary categorical variable for that patient, effectively converting unstructured free-text data into a structured format amenable to statistical analysis.

### Statistical analysis

For model development and validation, the total cohort was randomly split into a derivation cohort (n = 40,000; 57%) and a validation cohort (n = 30,597; 43%). Continuous variables were expressed as mean ± standard deviation, and categorical variables as number (percentage). Baseline characteristics between groups were compared using the Student's t-test for continuous variables and the Chi-squared (χ^2^) test for categorical variables.

A multivariable logistic regression model was developed on the derivation cohort to identify independent predictors of poor image quality. The dependent variable was image quality, dichotomised as poor versus adequate/good. Independent variables entered in the model included inpatient status, presence of a permanent pacemaker, prior cardiac surgery, history of any lung disease, clinical suspicion of heart failure, and the presence of any arrhythmia. Model fit was assessed using the Hosmer-Lemeshow test, and the explanatory power was evaluated using the Nagelkerke R^2^ value. The discriminative ability of the final model was quantified by the area under the receiver operating characteristic curve (AUC). All results are presented as odds ratios (OR) with corresponding 95% confidence intervals (CI). A two-sided *P*-value < 0.05 was considered statistically significant.

All data were tabulated and visualised using Microsoft Excel. Statistical analyses were performed using MedCalc^®^ Statistical Software version 23.2.8 (MedCalc Software Ltd, Ostend, Belgium), and final graphs were generated using OriginPro (OriginLab Corporation, Northampton, MA, USA).

### Cost-effectiveness analysis

To evaluate the potential economic impact of our predictive model, a cost-effectiveness analysis was conducted from the perspective of the healthcare provider. We simulated two clinical pathways for the entire study cohort (n = 70,597): (i) the current standard of care, and (ii) a model-guided triage strategy. The analysis was based on the 2024/25 UK National Health Service Payment Scheme tariffs (standard echocardiogram: £93; contrast-enhanced echocardiogram: £111; non-contrast cardiac magnetic resonance [CMR]: £448; and contrast-enhanced CMR: £639). It was assumed that any standard echocardiogram with poor image quality required a follow-up imaging study to be diagnostically useful. We modelled that of these poor-quality studies, 70% would proceed to a contrast-enhanced echocardiogram, while the remaining 30% would be referred for a definitive CMR (20% non-contrast and 10% contrast-enhanced). These assumptions were applied consistently across both simulated pathways.

## Results

### Baseline characteristics and image quality

The final analytical cohort comprised 70,597 patients. A substantial proportion of the studies, 34.3% (n = 24,213), were classified as having poor image quality. In contrast, 65.7% (n = 46,384) of scans were deemed to be of adequate or good quality. A sub-analysis revealed that studies with no diagnostic utility constituted 12.2% (n = 8582) of the total cohort, while those with limited diagnostic information accounted for 22.1% (n = 15,631). Among the diagnostically acceptable scans, 43.9% (n = 30,960) of the total cohort were rated as adequate, and 21.8% (n = 15,424) were rated as good.

As detailed in Table 1, patients with poor-quality echocardiograms were significantly more likely to be inpatients (27% vs. 19%, *p* < 0.0001) and were slightly younger on average (63.0 vs. 64.3 years, *p* < 0.0001); however, this age difference, while statistically significant, is unlikely to be clinically meaningful. The poor-quality group had a higher prevalence of non-sinus rhythm (31% vs. 26%, *p* < 0.0001), permanent pacemakers (3% vs. 2%, *p* < 0.0001), prior cardiac surgery (5% vs. 4%, *p* < 0.0001), and heart failure (5% vs. 2%, *p* < 0.0001). There was no significant difference in sex or body mass index between the two groups.

**Table 1.** Study demographics stratified by quality of transthoracic echocardiography imaging.

Variable (unit)	Poor diagnostic quality n = 24,213	Good diagnostic quality n = 46,384	*P*-value
Inpatient Requests	6511 (27%)	8817 (19%)	<0.0001^#^
Age at scan (years)	63.0 ± 17.2	64.3 ± 17.0	<0.0001^†^
Female sex	11,391 (47%)	21,860 (47%)	0.83^#^
Body mass index (kg m^−2^)	28.3 ± 5.8	28.2 ± 5.7	0.33^†^
Sinus rhythm	16,792 (69%)	34 209 (74%)	<0.0001^#^
Permanent pacemaker in situ	786 (3%)	1016 (2%)	<0.0001^#^
Diabetes mellitus	49 (0%)	51 (0%)	0.002^#^
History of myocardial infarction	326 (1%)	564 (1%)	0.14^#^
Previous cardiac surgery	1308 (5%)	2059 (4%)	<0.0001^#^
Heart failure	1242 (5%)	1151 (2%)	<0.0001^#^
Transcatheter aortic valve implantation	48 (0%)	66 (0%)	0.08^#^
Cardiac transplant recipient	4 (0%)	7 (0%)	0.89^#^
Repaired aortic aneurysm	6 (0%)	6 (0%)	0.25^#^
Aortic stenosis	1197 (5%)	2107 (5%)	0.02^#^
Mitral valve repair	104 (0%)	350 (1%)	<0.0001^#^
Mitral stenosis	52 (0%)	78 (0%)	0.17^#^

Data are mean ± standard deviation or number (percentage).

†Student *t*-test for continuous variables; #χ^2^ test for categorical variables.

### Derivation and validation cohorts

The baseline characteristics of the derivation (n = 40,000) and validation (n = 30,597) cohorts are presented in Table 2. These cohorts were used for model development and independent validation of the prediction model.

**Table 2.** Differences in the clinical characteristics of the derivation and validation cohorts.

Variable (unit)	Derivation cohort	Validation cohort	*P*-value
N	40,000 (57%)	30,597 (43%)	—
Poor diagnostic quality	13,999 (35%)	10,214 (33%)	<0.0001
In-patient status	4916 (12%)	10,412 (34%)	<0.0001
Age at scan (years)	66.8 ± 14.9	60.0 ± 18.8	<0.0001
Female sex	19,590 (49%)	13,661 (45%)	<0.0001
Body mass index (kg m^−2^)	28.4 ± 5.7	28.1 ± 5.9	<0.0001
Permanent pacemaker in situ	1207 (3%)	595 (2%)	<0.0001
Diabetes mellitus	68 (0%)	32 (0%)	0.0220
History of myocardial infarction	466 (1%)	424 (1%)	0.0092
Previous cardiac surgery	2191 (5%)	1176 (4%)	<0.0001
Transcatheter aortic valve implantation	75 (0%)	39 (0%)	0.0490
Repaired tetralogy of Fallot	79 (0%)	27 (0.1%)	0.0002
Repaired atrial septal defect	66 (0%)	32 (0%)	0.0326
Aortic regurgitation	567 (1%)	289 (1%)	<0.0001
Other congenital heart disease	32 (0.1%)	46 (0%)	0.0053
Mitral regurgitation	768 (2%)	359 (1%)	<0.0001

### Predictors of poor image quality in the derivation cohort

In the multivariable logistic regression analysis performed on the derivation cohort, several factors emerged as significant independent predictors of poor echocardiographic image quality (Figure 1). The strongest predictor was a history of any lung disease (OR 2.04, 95% CI 1.59–2.61, *p* < 0.0001), followed by a clinical suspicion of heart failure (OR 1.87, 95% CI 1.68–2.07, *p* < 0.0001). An inpatient scan setting also significantly increased the odds of poor image quality (OR 1.67, 95% CI 1.57–1.77, *p* < 0.0001). Other significant predictors included prior cardiac surgery (OR 1.23, 95% CI 1.12–1.34, *p* < 0.0001), the presence of any arrhythmia (OR 1.23, 95% CI 1.17–1.30, *p* < 0.0001), and having a permanent pacemaker (OR 1.20, 95% CI 1.07–1.36, *p* = 0.003) (Figure 2). The overall model demonstrated a good fit (Hosmer-Lemeshow *P* = 0.1090) but had modest discriminative power, with an AUC of 0.56 (95% CI 0.557–0.567) and a Nagelkerke R^2^ of 0.020.

### Model validation

The predictive model derived from the derivation cohort was subsequently tested on a separate validation cohort (n = 30,597). The model's performance was consistent with the initial findings, demonstrating a statistically significant, albeit modest, ability to discriminate between poor and good quality scans. The AUC in the validation cohort was 0.580 (95% CI 0.572 to 0.583, *p* < 0.0001). The Youden index identified an optimal probability cut-off of >0.6, which yielded a sensitivity of 67.3% and a specificity of 45.7% for predicting poor image quality. At this threshold, the overall accuracy of the model was 60.1%.

### Cost-effectiveness analysis

In the economic model, the use of the prediction tool (Figure 3) to triage patients at high risk of poor acoustic windows led to an overall reduction in imaging costs compared with the current pathway (Table 3). Using 2024/25 NHS tariffs, the total cost for the current pathway was £12,163,543 versus £11,846,212 for the model-guided triage pathway, representing a net saving of £317,331 across 70,597 studies (≈£4.5 per study). These savings arise primarily from avoiding low yield “standard first → non-diagnostic → second test” sequences. High-risk patients are instead directed to contrast echocardiography as the first-line test (or, in a small minority, directly to CMR), thereby reducing the need for repeat imaging. Importantly, this strategy does not involve withholding echocardiography from any subgroup, including inpatients. Rather, it supports more appropriate first-test selection, ensuring that patients who are predicted to have poor windows receive contrast echocardiography upfront. In practice, this aligns with a pragmatic approach in which sonographers can switch to contrast during the same inpatient session when initial image quality is suboptimal, supported by ready departmental access to contrast.

**Table 3.** Cost-Effectiveness analysis of clinical pathways.

Pathway & investigation	Number of patients	Cost per patient (£)	Total cost (£)
Scenario 1: Current Standard of Care			
Initial Standard Echocardiogram	70,597	93	6,565,521
*Follow-up for Poor Quality Scans (n* *=* *24,213)*			
Contrast Echocardiogram (70%)	16,949	111	1,881,339
Non-Contrast CMR (20%)	4843	448	2,169,664
Contrast CMR (10%)	2421	639	1,547,019
Total Cost (Standard of Care)			12,163,543
Scenario 2: Model-Guided Triage			
Low-Risk Arm (n = 19,055)			
Initial Standard Echocardiogram	19,055	93	1,772,115
*Follow-up for False Negatives (n* *=* *8189)*			
Contrast Echocardiogram (70%)	5732	111	636,252
Non-Contrast CMR (20%)	1638	448	733,824
Contrast CMR (10%)	819	639	523,341
High-Risk Arm (n = 51,542)			
Initial Contrast Echocardiogram	51,542	111	5,721,162
*Follow-up for True Positives (n* *=* *16,024)*			
Non-Contrast CMR (20%)	3205	448	1,435,840
Contrast CMR (10%)	1602	639	1,023,678
Total Cost (Model-Guided Triage)			11,846,212
Total Cost Saving			£317,331

## Discussion

### Statement of principal findings

In this large retrospective cohort of 70,597 real-world transthoracic echocardiograms, one-third of studies had limited or non-diagnostic image quality. Lung disease was the strongest independent predictor, followed by suspected heart failure, inpatient status, arrhythmia, prior cardiac surgery and permanent pacemaker presence. Although discrimination was modest, the model remained operationally useful in a pragmatic triage simulation by reducing downstream imaging costs.

### Strengths and weaknesses of the study

Strengths include the large consecutive dataset, routine real-world case mix, separate derivation and validation cohorts, and the use of a reproducible rule-based NLP pipeline to extract indications from free-text reports. The study also addresses a service-delivery question of direct relevance to echocardiography laboratories. Limitations include the retrospective single-centre design, modest discriminative ability (AUC 0.56–0.58), routine sonographer categorisation rather than core-laboratory adjudication, and incomplete capture of operator identifiers, patient positioning, machine metadata, transducer selection and advanced imaging settings. Lung disease was also captured as a binary umbrella variable without reliable subtype or severity data.

### Strengths and weaknesses in relation to other studies, discussing particularly any differences in results

Our findings are broadly consistent with previous work showing that inpatient status and greater clinical complexity are associated with poorer echocardiographic yield and more frequent need for contrast agents.^15,16^ Unlike some earlier cohorts, however, we did not observe a meaningful association for sex or body mass index.^15^ Differences in case mix, operational definitions of poor image quality and routine reporting practice may explain this. Beyond pulmonary comorbidity itself, thoracic geometry may also be relevant. Recent studies suggest that chest wall conformation can affect reproducibility of left ventricular ejection fraction and global longitudinal strain, while a systematic review of pectus excavatum supports the importance of chest wall morphology when interpreting echocardiographic measurements.^17,18^

### Meaning of the study: Possible mechanisms and implications for clinicians or policymakers

Taken together, these observations support a broader thoracic phenotype framework in which pulmonary hyperinflation, altered chest wall geometry, postoperative change, rhythm irregularity and device-related artefact can collectively impair acoustic windows. The practical implication is not to withhold echocardiography from higher-risk patients, but to improve first-test selection. Patients with a high pre-test probability of poor image quality may benefit from planned use of contrast echocardiography or, where clinically appropriate, earlier referral to alternative imaging. Even with modest discrimination, a pre-acquisition triage tool may improve workflow efficiency and reduce repeat testing at scale.

### Unanswered questions and future research

External validation is required in centres with different referral patterns, operator experience and equipment. Future studies should incorporate operator-level factors, machine metadata and more structured respiratory and thoracic phenotyping, including chest wall conformation and objective pulmonary function where available. Prospective studies should also assess whether combining simple clinical pre-test factors with point-of-acquisition artificial intelligence-based image-quality assessment can further improve pathway selection.

## Conclusions

Several routinely available clinical factors independently influence transthoracic echocardiographic image quality. Recognising these factors before image acquisition may help direct higher-risk patients towards contrast echocardiography or alternative imaging, thereby reducing repeat testing and improving pathway efficiency.

## Abbreviations

AUC: Area Under Curve
BSE: British Society of Echocardiography
CI: Confidence Intervals
CMR: Cardiac Magnetic Resonance
CVD: Cardiovascular Disease
NLP: Natural Language Processing
OR: Odds Ratios

## References

[1] Roth GA Mensah GA Johnson CO , et al. Global burden of cardiovascular diseases and risk factors, 1990–2019: update from the GBD 2019 study. J Am Coll Cardiol 2020; 76: 2982–3021. 33309175 10.1016/j.jacc.2020.11.010 PMC7755038

[2] McAloon CJ Boylan LM Hamborg T , et al. The changing face of cardiovascular disease 2000–2012: an analysis of the world health organisation global health estimates data. Int J Cardiol 2016; 224: 256–264. 27664572 10.1016/j.ijcard.2016.09.026

[3] Martin SS Aday AW Almarzooq ZI , et al. 2024 heart disease and stroke statistics: a report of US and global data from the American Heart Association. Circulation 2024; 149: e347–e913. 10.1161/CIR.0000000000001209 PMC12146881 38264914

[4] McDonagh TA Metra M Adamo M , et al. 2021 ESC guidelines for the diagnosis and treatment of acute and chronic heart failure. Eur Heart J 2021; 42: 3599–3726. 34447992 10.1093/eurheartj/ehab368

[5] Vahanian A Beyersdorf F Praz F , et al. 2021 ESC/EACTS guidelines for the management of valvular heart disease. Eur Heart J 2022; 43: 561–632. 34453165 10.1093/eurheartj/ehab395

[6] Adler Y Charron P Imazio M , et al. 2015 ESC guidelines for the diagnosis and management of pericardial diseases: the task force for the diagnosis and management of pericardial diseases of the European Society of Cardiology (ESC)Endorsed by: the European association for cardio-thoracic surgery (EACTS). Eur Heart J 2015; 36: 2921–2964. 26320112 10.1093/eurheartj/ehv318 PMC7539677

[7] Ferraz S Coimbra M Pedrosa J . Assisted probe guidance in cardiac ultrasound: a review. Front Cardiovasc Med 2023; 10: 1056055. 36865885 10.3389/fcvm.2023.1056055 PMC9971589

[8] Nolan MT Thavendiranathan P . Automated quantification in echocardiography. JACC Cardiovasc Imaging 2019; 12: 1073–1092. 31171260 10.1016/j.jcmg.2018.11.038

[9] Lancellotti P Płońska-Gościniak E Garbi M , et al. Cardiovascular imaging practice in Europe: a report from the European association of cardiovascular imaging. Eur Heart J Cardiovasc Imaging 2015; 16: 697–702. 25944050 10.1093/ehjci/jev116

[10] Shaw LJ . Impact of contrast echocardiography on diagnostic algorithms: pharmacoeconomic implications. Clin Cardiol 1997; 20: I39–I48. 10.1002/clc.4960201309 PMC6656058 9383601

[11] Bovenkamp AA van de Enait V Man FS de , et al. Validation of the 2016 ASE/EACVI guideline for diastolic dysfunction in patients with unexplained dyspnea and a preserved left ventricular ejection fraction. J Am Heart Assoc 2021; 10: e021165. 10.1161/JAHA.121.021165 PMC8649534 34476984

[12] Hua A McCaughan V Wright M , et al. Appropriateness, diagnostic value, and outcomes of repeat testing following index echocardiography. Echocardiogr Mt Kisco N 2018; 35: 24–29. 10.1111/echo.13726 28994195

[13] Pack QR Priya A Lagu T , et al. Association between inpatient echocardiography use and outcomes in adult patients with acute myocardial infarction. JAMA Intern Med 2019; 179: 1176–1185. 31206134 10.1001/jamainternmed.2019.1051 PMC6580445

[14] Pathan F Fonseca R Marwick TH . Usefulness of hand-held ultrasonography as a gatekeeper to standard echocardiography for ‘rarely appropriate’ echocardiography requests. Am J Cardiol 2016; 118: 1588–1592. 27810098 10.1016/j.amjcard.2016.08.027

[15] Ellenberger K Jeyaprakash P Sivapathan S , et al. The effect of obesity on echocardiographic image quality. Heart Lung Circ 2022; 31: 207–215. 34373191 10.1016/j.hlc.2021.06.525

[16] Fraiche AM Manning WJ Nagueh SF , et al. Identification of need for ultrasound enhancing agent study (the IN-USE study). J Am Soc Echocardiogr Off Publ Am Soc Echocardiogr 2020; 33: 1500–1508. 10.1016/j.echo.2020.07.015 PMC7722181 32919859

[17] Sonaglioni A Nicolosi GL Granato A , et al. Influence of chest wall conformation on reproducibility of main echocardiographic indices of left ventricular systolic function. Minerva Cardiol Angiol 2024; 72: 111–124. 38231080 10.23736/S2724-5683.23.06475-X

[18] Sonaglioni A Fagiani V Nicolosi GL , et al. The influence of pectus excavatum on biventricular mechanics: a systematic review and meta-analysis. Minerva Cardiol Angiol. Epub ahead of print 2024. DOI: 10.23736/S2724-5683.24.06614-6. 39315893

